# Organic and Functional Nervous Diseases

**Published:** 1907-05

**Authors:** 


**Organic and Functional Nervous Diseases.** By **M. Allen Starr,** M.D., Ph.D., LL.D., Professor of Neurology in the College of Physicians and Surgeons, New York; ex-President of the American Neurological Association and of the New York Neurological Society; Corresponding Member of the Societe de Neurologie de Paris, etc. Octavo, 824 pages with 284 engravings and 26 full-page plates in colors and monochrome. Philadelphia: F. A. Davis Company. (Price, Cloth, $6.00, net; leather, $7.00, net.)

The first edition of Dr. Starr's book met with such favorable recognition from the medical profession as to exhaust the supply within a year. This new book is marked by a careful revision of the first on organic nervous diseases, and adds a new section covering functional nervous diseases. The work is really the analytical study of Dr. Starr's large hospital and private practice, and this book gives the author's views and emphasises his personal observation and experience in the presentation of each subject. While he does not neglect reference to the views and theories of other authors, still very little is noted in this book which does not harmonise with his views.

Dr. Starr has seen fit to exclude many diseases which most authors include in their works on diseases of the nervous system,—notably, hydrophobia, trophic disorders such as lipomatosis, megalocephaly, scleroderma, and trophoderma, so that many will be disappointed in not obtaining the author's views on these diseases. We agree with him that as works upon general medicine now include a complete description of these subjects, it is unnecessary to deal with them in a treatise like this.

A study of this book leads us to note the author's exact methods in diagnosis, and his marked advocacy of the surgical treatment of many of the nervous affections. There is no book on the subject which has appeared of late years that deals more directly with the treatment, surgical and medical, of nervous diseases than Dr. Starr's. It is beautifully printed and illustrated. We cordially recommend it to students of this specialty as one of the best of modern neurological works.

